# A SCID Mouse Model To Evaluate the Efficacy of Antivirals against SARS-CoV-2 Infection

**DOI:** 10.1128/jvi.00758-22

**Published:** 2022-08-04

**Authors:** Rana Abdelnabi, Caroline S. Foo, Suzanne J. F. Kaptein, Robbert Boudewijns, Laura Vangeel, Steven De Jonghe, Dirk Jochmans, Birgit Weynand, Johan Neyts

**Affiliations:** a KU Leuven Department of Microbiology, Immunology and Transplantation, Rega Institute for Medical Research, Laboratory of Virology and Chemotherapy, Leuven, Belgium; b The VirusBank, Leuven, Belgium; c Global Virus Network, Baltimore, Maryland, USA; d KU Leuven Department of Imaging and Pathology, Division of Translational Cell and Tissue Research, Leuven, Belgium; Cornell University

**Keywords:** SARS-CoV-2, mouse model, Beta variant, antivirals, nirmatrelvir, molnupiravir

## Abstract

Ancestral severe acute respiratory syndrome coronavirus 2 (SARS-CoV-2) lacks the intrinsic ability to bind to the mouse ACE2 receptor, and therefore establishment of SARS-CoV-2 mouse models has been limited to the use of mouse-adapted viruses or genetically modified mice. Interestingly, some of the variants of concern, such as the Beta B.1.351 variant, show an improved binding to the mouse receptor and hence better replication in different wild-type (WT) mouse species. Here, we describe the establishment of a SARS-CoV-2 Beta B.1.351 variant infection model in male SCID mice as a tool to assess the antiviral efficacy of potential SARS-CoV-2 small-molecule inhibitors. Intranasal infection of male SCID mice with 10^5^ 50% tissue culture infective doses (TCID_50_) of the Beta B.1.351 variant resulted in high viral loads in the lungs and moderate signs of lung pathology on day 3 postinfection. Treatment of infected mice with the antiviral drugs molnupiravir (200 mg/kg, twice a day [BID]) or nirmatrelvir (300 mg/kg, BID) for 3 consecutive days significantly reduced the infectious virus titers in the lungs by 2 and 3.9 log_10_ TCID_50_/mg of tissue, respectively, and significantly improved lung pathology. Together, these data demonstrate the validity of this SCID mouse Beta B.1.351 variant infection model as a convenient preclinical model for assessment of potential activity of antivirals against SARS-CoV-2.

**IMPORTANCE** Unlike the ancestral SARS-CoV-2 strain, the Beta (B.1.351) variant of concern has been reported to replicate to some extent in WT mice (C57BL/6 and BALB/c). We demonstrate here that infection of SCID mice with the Beta variant resulted in high viral loads in the lungs on day 3 postinfection. Treatment of infected mice with molnupiravir or nirmatrelvir for 3 consecutive days markedly reduced the infectious virus titers in the lungs and improved lung pathology. The SARS-CoV2 SCID mouse infection model, which is ideally suited for antiviral studies, offers an advantage in comparison to other SARS-CoV2 mouse models, in that there is no need for the use of mouse-adapted virus strains or genetically modified mice. Mouse models also have advantages over hamster models because (i) lower amounts of test drugs are needed, (ii) more animals can be housed in a cage, and (iii) reagents to analyze mouse samples are more readily available than those for hamsters.

## INTRODUCTION

Since its emergence in China at the end of 2019, the severe acute respiratory syndrome coronavirus 2 (SARS-CoV-2) has resulted in a global pandemic with officially >517 million cases (as of 10 May 2022) and ≈15 million deaths, as estimated by WHO (1). Several SARS-CoV-2 variants of concern (VoCs) that result in immune escape and/or enhanced viral transmission have since emerged (2, 3). Small animal models are necessary to study the virus-induced pathogenesis as well as to serve as preclinical tool to assess the efficacy of vaccines and therapeutics against the viral infection. Similar to SARS-CoV, SARS-CoV-2 enters host cells through attachment to cellular angiotensin-converting enzyme 2 (ACE2) (4). Since SARS-CoV-2 binds efficiently to the hamster ACE2 (5), Syrian hamsters are considered one of the best small animal models available for SARS-CoV-2. On the other hand, the spike protein of the ancestral SARS-CoV-2 strain lacks the intrinsic ability to efficiently bind to the murine ACE2 (5), and hence this strain has limited replication in wild-type (WT) mice. Consequently, alternative strategies have been developed to allow the establishment of mouse models for SARS-CoV-2. One of these strategies is adaptation of the virus in murine lung tissues to enhance binding capacity to the murine ACE2 (6, 7). Other strategies focused on introduction of human ACE2 in wild-type mice either by transduction of adenovirus or an adeno-associated virus that expresses human ACE2 (8) or by using genetically modified human ACE2 transgenic (9) or humanized mice (10). Unlike the ancestral strain, some of the evolved SARS-CoV-2 VoCs proved to carry spike protein mutations, mainly the mutation cause by an N-to-Y change at position 501 (N501Y), that enable efficient binding to the murine ACE2 and hence better replication in WT mice (11, 12). Besides the N501Y mutation, the spike of the Beta B.1.135 variant carries the K417N mutation, which was previously reported in a virulent mouse-adapted SARS-CoV-2 variant (13). Several studies have shown the ability of the Beta variant to replicate to some extent in WT mouse strains such as C57BL/6 (11, 12) and BALB/c (14, 15). Here, we wanted to explore whether the Beta SARS-CoV-2 variant replicates more efficiently in severe combined immune deficient (SCID) mice than in wild-type mice and whether, in such a case, SCID mice can be used to develop a sufficiently robust infection model to study the efficacy of small-molecule inhibitors of SARS-CoV-2 infection.

## RESULTS

**FIG 1:**
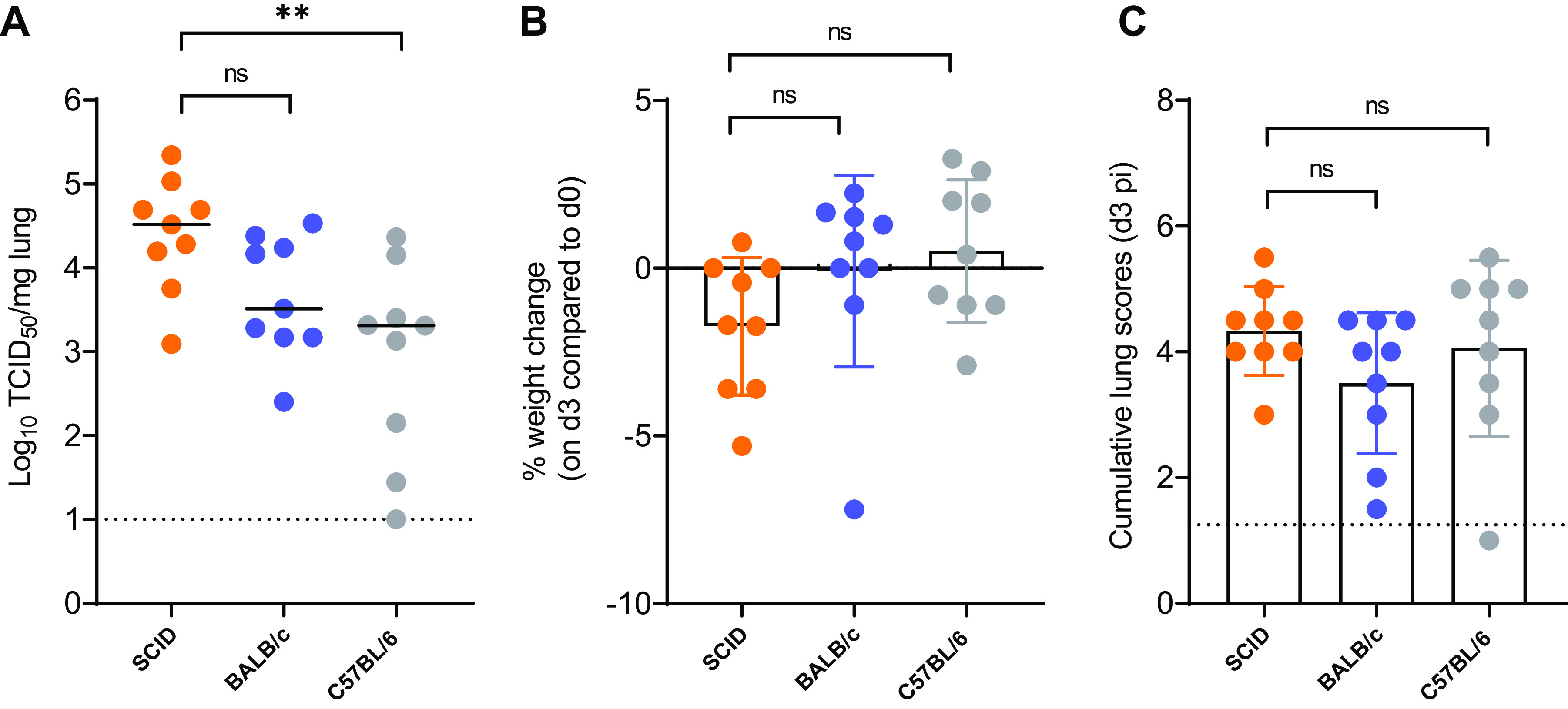
Replication of Beta (B.1.351) SARS-CoV-2 in different mouse strains. (A) Infectious viral titers in the lungs of male SCID, male BALB/c, and male C57BL/6 mice infected with 10^5^ TCID_50_ of Beta SARS-CoV-2 variants at 3 days p.i. are expressed in log_10_ TCID_50_ per milligram of lung tissue. Individual data and median values are presented. (B) Weight change at day 3 p.i. as a percentage, normalized to the body weight at the time of infection. Bars represent means ± standard deviations (SD). (C) Cumulative severity score from H&E-stained slides of lungs from infected mice at day 3 p.i. Individual data are presented, and bars represent means ± SD. The dotted line represents the mean score of untreated noninfected mice. Data were analyzed with the Kruskal-Wallis test. ns, nonsignificant; **, *P* < 0.01. Data are from two independent experiment with *n* = 9 per group, d3 pi = day 3 postinfection.

First, a small pilot study was performed to assess the efficiency of replication of the Beta (B.1.351) SARS-CoV-2 variant in male SCID mice in comparison to replication in immunocompetent male BALB/c and C57BL/6 mice. All mice (*n* = 9 per strain) were infected with 10^5^ 50% tissue culture infective doses (TCID_50_) of the Beta variant. Based on previous studies where the Beta variant was used to infect wild-type mice (14–16), we selected day 3 postinfection (p.i.) as the endpoint at which all animals were euthanized and lungs were collected to quantify the infectious virus titers. The infectious virus titers in the lungs of infected SCID mice (median 3.28 × 10^4^ TCID_50_/mg lung tissue) was markedly higher than that observed in the lungs of infected BALB/c mice (median 3.26 × 10^3^ TCID_50_/mg lung tissue; *P* = 0.22 [nonsignificant]) and C57BL/6 mice (median 2.05 × 10^3^ TCID_50_/mg lung tissue; *P* = 0.0098) (Fig. 1A). A minor weight loss was observed on the sacrifice day for infected SCID and BALB/c mice (average body weight change of −1.7% and −0.09%, respectively) but not for infected C57BL/6 mice (average body weight change of 0.5%) (Fig. 1B). Histological examination of lungs from all infected mice showed some signs of bronchopneumonia, peribronchial inflammation, and perivascular inflammation (Fig. 1C). The average cumulative lung pathology scores were 4.3, 3.5, and 4 for infected SCID, BALB/c, and C57BL/6 mice, respectively (Fig. 1C).

**FIG 2:**
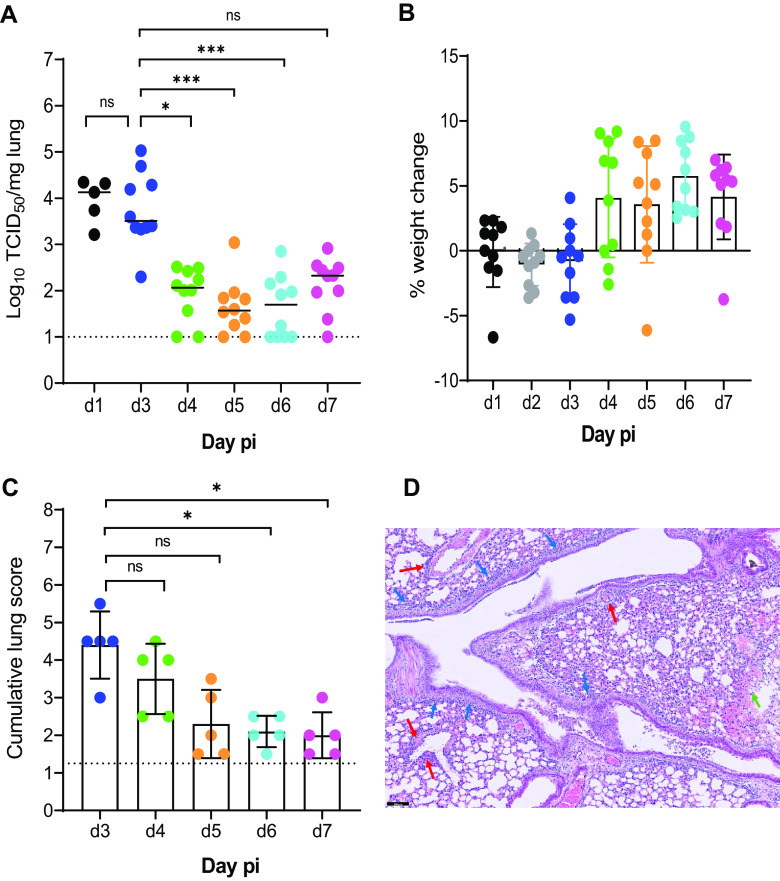
Replication kinetics of Beta (B.1.351) SARS-CoV-2 in male SCID mice. (A) Infectious viral loads in the lungs of male SCID mice infected with 10^5^ TCID_50_ of Beta SARS-CoV-2 variants at different days postinfection were expressed as the log_10_ TCID_50_ per milligram of lung tissue. Individual data and median values are presented. (B) Weight change at different days postinfection as a percentage, normalized to the body weight at the time of infection. Bars represent means ± SD. (C) Cumulative severity score from H&E-stained slides of lungs from infected mice at different days postinfection. Individual data are presented, and bars represent means ± SD. The dotted line represents the mean score of untreated noninfected mice. (D) Representative H&E image of lung from SCID mouse infected with the Beta variant at day 3 p.i., showing limited peribronchial inflammation (blue arrows), significant perivascular inflammation (red arrows), and intra-alveolar hemorhage (green arrow). Bar, 100 μm. Data were analyzed with the Kruskal-Wallis test. ns, nonsignificant; *, *P* < 0.05; ***, *P* < 0.001. All data are from 2 independent experiments with 10 animals per group, except for day 1 data in panel A and the data in panel C.

Next, we explored the kinetics of replication of the Beta variant in SCID mice. To that end, 7- to 9-week-old male SCID mice were infected intranasally with 10^5^ TCID_50_ of the Beta variant. Five mice were euthanized at day 1 p.i., and then from days 3 through 7 p.i., 10 animals were euthanized per time point and lungs were collected to quantify the infectious virus titers. The highest infectious virus titers were observed at day 1 and day 3 p.i. (Fig. 2A). From day 4 p.i. onwards, the infectious virus titers in the lungs were significantly lower than those observed at day 3 p.i. (Fig. 2A). A minor weight loss was observed from day 1 to day 3 p.i. (average body weight change of −0.09% to −1.1%), after which animals started to gain weight normally (average body weight change of 4% on day 4 p.i.) (Fig. 2B). When a group of 5 infected mice were monitored up to 14 days p.i., no weight loss or any signs of morbidity were observed in this group (average body weight change of 13% on day 14 p.i.). Histological examination of lungs from infected mice showed the highest pathology score on day 3 p.i., with a median cumulative pathology score of 4.5, and pathology scores significantly dropped from day 6 p.i. (Fig. 2C). Hematoxylin and eosin (H&E)-stained images of the lungs from infected mice at day 3 p.i. revealed mild signs of peribronchial inflammation, significant perivascular inflammation, and intra-alveolar hemorrhage (Fig. 2D).

**FIG 3:**
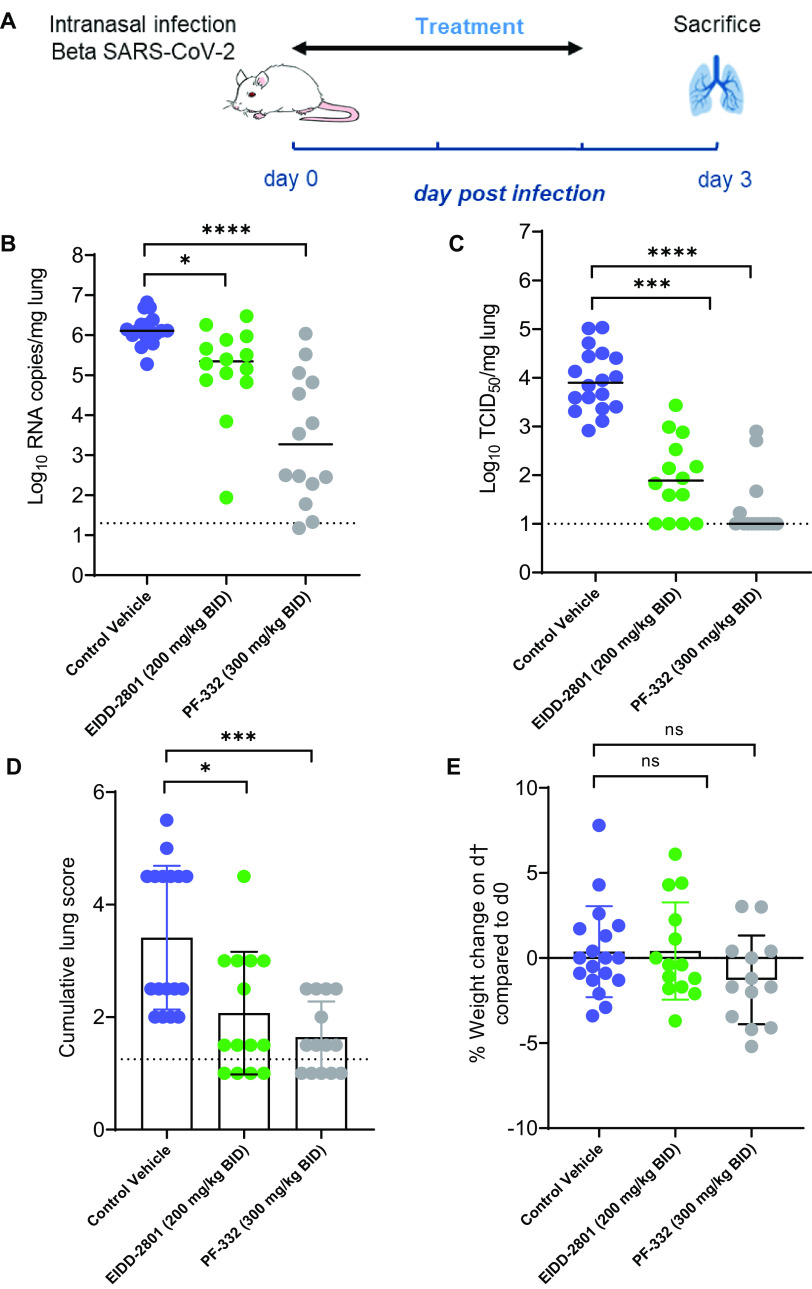
Molnupiravir (EIDD-2801) and nirmatrelvir (PF-332) reduced viral loads in the lungs of Beta (B.1.351) SARS-CoV-2–infected SCID mice. (A) Setup of the study. (B) Viral RNA levels in the lungs of control (vehicle-treated), EIDD-2801-treated (200 mg/kg, BID), and PF-332-treated (300 mg/kg, BID) SCID mice infected with SARS-CoV-2 (B.1.351) at day 3 p.i. are expressed as log_10_ SARS-CoV-2 RNA copies per milligram of lung tissue. Individual data and median values are presented. (C) Infectious viral loads in the lungs of control (vehicle-treated), EIDD-2801-treated, and PF-332-treated beta SARS-CoV-2−infected SCID mice at day 3 p.i. are expressed as the log_10_ TCID_50_ per milligram of lung tissue. Individual data and median values are presented. (D) Cumulative severity scores from H&E-stained slides of lungs from control (vehicle-treated), EIDD-2801-treated (200 mg/kg, BID), and PF-332-treated (300 mg/kg, BID) SARS-CoV-2−infected SCID mice at day 3 p.i. Individual data are presented, and bars represent means ± SD. The dotted line represents the mean score of untreated noninfected mice. (E) Weight change at day 3 p.i. as a percentage, normalized to the body weight at the time of infection. Bars represent means ± SD. Data were analyzed with the Kruskal-Wallis test. *, *P* < 0.05; ***, *P* < 0.001; ****, *P* < 0.0001; ns, nonsignificant. All data are from two independent experiments with 14 animals per group, except for the vehicle group (*n* = 18).

If the infectious virus detected at day 3 postinfection represented actively replicating virus, it should have been possible to suppress replication by treating the animals with antiviral drugs. We therefore assessed the potential antiviral efficacy of two clinically relevant SARS-CoV-2 inhibitors, i.e., molnupiravir (EIDD-2801) and nirmatrelvir (PF-332), against Beta variant replication in SCID mice. Briefly, male SCID mice were treated twice daily by oral gavage with either vehicle, molnupiravir (200 mg/kg), or nirmatrelvir (300 mg/kg) for three consecutive days, starting from the day of infection with the Beta variant (Fig. 3A). Mice were euthanized at day 3 p.i. for collection of lung tissues. A significant reduction of viral RNA loads was observed in the molnupiravir-treated (0.8 log_10_ genome copies/mg tissue; *P* = 0.011) and nirmatrelvir-treated (2.8 log_10_ genome copies/mg tissue; *P* < 0.0001) groups compared to the vehicle control (Fig. 3B). Moreover, treatment of mice with molnupiravir and nirmatrelvir significantly reduced the infectious virus titers in the lungs by 2 log_10_ (*P* = 0.0001) and 3.9 log_10_ (*P* < 0.0001) TCID_50_/mg tissue, respectively, compared to the vehicle-treated group (Fig. 3C). No infectious virus titers were detected in 4 (out of 14) and 8 (out of 14) animals in the molnupiravir- and nirmatrelvir-treated groups, respectively (Fig. 3C). A significant improvement of lung histopathology scores was also observed in both the molnupiravir-treated (*P* = 0.018) and nirmatrelvir-treated (*P* = 0.0005) groups (Fig. 3D). No significant weight loss or clinical signs of adverse effects were observed in the compound-treated groups (Fig. 3E).

## DISCUSSION

The emergence of SARS-CoV-2 VoCs has raised a lot of concern as these variants displayed the ability to escape vaccine-induced or naturally acquired immunity and to transmit faster than the ancestral strains. In addition, some of these variants have acquired certain mutations in the spike protein that allow them to expand their host species (2, 12). The Beta variant (B.1.351 or 501Y.V2) was first reported in South Africa in October 2020 (17). The Beta variant has acquired three mutations in the receptor binding domain (RBD), namely, N501Y, K417N, and E484K, in addition to other mutations in the spike and nonstructural proteins (2). Among these mutations, the N501Y mutation (also present in the alpha variant) has been previously described in mouse-adapted viruses and proven to play an important role in increasing the affinity to the mouse ACE2 receptor (6). The K417N mutation has also previously been reported in a virulent mouse-adapted SARS-CoV-2 variant (13). In a pseudotype-based entry assay, the pseudoviruses carrying the Beta variant of the spike protein attached more efficiently to the mouse ACE2 receptor than the alpha variants, suggesting that the K417N and E484K mutations in the RBD of the Beta variant may further enhance the binding to the mouse receptor (11). Recently, a comparative infection study in BALB/c mice revealed that the Beta variant replicates more efficiently than the alpha or delta variant (15).

Here, we wanted to assess the infectivity of the Beta SARS-CoV-2 variant in an immunodeficient mouse model, i.e., SCID mice, with the aim to develop a robust SARS-CoV-2 mouse infection model for preclinical evaluation of potential antivirals. So far, the hamster SARS-CoV-2 infection model has been regarded as the best model to study the effect of antiviral agents, yet use of mice would facilitate such studies. We selected SCID mice, as these animals are severely deficient in functional B and T lymphocytes and, therefore, they are believed to be more susceptible to viral infections than immunocompetent mice. Indeed, in our pilot infection study, the infectious virus titers of the Beta variant in the lungs of infected SCID mice on day 3 p.i. were significantly higher than that observed in the lungs of immunocompetent BALB/c (1 log_10_ higher) or C57BL/6 mice (1.2 log_10_ higher) that were infected in parallel. Viral persistence in the lungs of SCID mice was observed in most of the infected animals up to 7 days p.i. However, the infectious virus titers dropped significantly beyond day 3 p.i. Therefore, day 3 p.i. was selected as the endpoint for antiviral testing.

Nirmatrelvir (PF-332; Pfizer) is a potent inhibitor of the main protease M^pro^ (or 3CL protease) of SARS-CoV-2 and other coronaviruses (18). Paxlovid (nirmatrelvir and ritonavir tablets, copackaged for oral use) has been authorized by the FDA and EMA as well as by other regions. Molnupiravir (Lagevrio; EIDD-2801; Merck) is the orally bioavailable prodrug of the ribonucleoside analog N4-hydroxycytidine (NHC; EIDD-1931), which was initially developed for influenza (19) and has now also been approved by several countries and regions for the treatment of COVID-19.

We previously showed that molnupiravir (EIDD-2801) and nirmatrelvir (PF-332) significantly inhibit the replication of the Beta variant in Syrian hamsters (20, 21). Therefore, we used these two antiviral drugs to validate the SCID mouse, Beta variant infection model for antiviral studies. Treatment of Beta variant-infected SCID mice for 3 consecutive days with molnupiravir (200 mg/kg, twice a day [BID]) or nirmatrelvir (300 mg/kg, BID) significantly reduced viral loads in the lungs of infected mice, with a potency close to that observed against the same variant in our Syrian hamster model (where the endpoint was at 4 days postinfection) (20, 21). An improvement in lung pathology scores was also observed in the molnupiravir- and nirmatrelvir-treated SCID mice compared to the vehicle-treated mice. Thus, the SCID mouse, Beta variant infection model may serve as a useful tool to assess the *in vivo* efficacy of antiviral molecules against SARS-CoV-2.

It is surprising that infected SCID mice seem to control the infection by day 4 postinfection. Moreover, monitoring a group of infected mice up to 14 days p.i. did not reveal any morbidity signs or weight loss over time. Typically, infection of SCID mice with viruses (those that are able to replicate in mice) results in a lethal infection (22–24).

The advantages of using this mouse model over other SARS-CoV-2 mouse models for initial *in vivo* evaluation of antivirals include the use of a real clinical isolate without the need to use mouse-adapted strains or genetically modified animals. Moreover, this mouse model has some advantages over bigger rodents, such as hamsters, as roughly 4- to 6-fold less of the test drug is needed for the *in vivo* efficacy studies (average weight of a hamster is 80 to 120 g, versus 20 g for mice), which will save a lot of material; this is of particular important in cases of highly priced or difficult-to-synthesize compounds. The more convenient housing conditions, as up to 5 mice can be cohoused in one cage, versus 2 hamsters per cage, are important for the capacity of the high-level biosafety animal facility. Finally, more reagents are available for analysis of mouse samples than for hamster ones. Consequently, such a model will enable testing more compounds in a shorter period of time. On the other hand, a limitation of this model is that, unlike for hamsters, mice are only susceptible to the Beta variant. Since small-molecule inhibitors should have equipotent activity against all variants, this will be of limited concern for studies with such drugs. However, for testing of therapeutic antibodies, infection models (in hamsters) with the different VoCs will still be needed. Likewise, for vaccine studies fully immunocompetent animals are needed; SCID mice are not useful for this purpose. Therefore, this SCID mouse, Beta variant infection model will be advantageous mainly for the evaluation of small-molecule inhibitors of SARS-CoV-2 replication.

## MATERIALS AND METHODS

### Virus.

The SARS-CoV-2 strain used in this study, the Beta variant B.1.351 (hCoV-19/Belgium/rega-1920/2021; EPI_ISL_896474, 2021-01-11), was recovered from a nasopharyngeal swab taken from a patient with respiratory symptoms returning to Belgium in January 2021 (25). A passage 2 virus on Vero E6 cells was used for the study described here. Live virus-related work was conducted in the high-containment A3 and biosafety level 3+ facilities of the KU Leuven Rega Institute (3CAPS) under licenses AMV 30112018 SBB 219 2018 0892 and AMV 23102017 SBB 219 20170589 according to institutional guidelines.

### Compounds.

Molnupiravir (EIDD-2801) was purchased from Excenen Pharmatech Co., Ltd. (China) and was formulated as a 50-mg/mL stock in a vehicle containing 10% polyethylene glycol 400 (Sigma) and 2.5% Kolliphor-EL (Sigma) in water. Nirmatrelvir (PF-332; from Wuxi, USA) was formulated as a 125-mg/mL stock in a vehicle containing 43% ethanol and 27% propylene glycol (Sigma) in sterile distilled water.

### Cells.

Vero E6 cells (African green monkey kidney; ATCC CRL-1586) were cultured in minimal essential medium (Gibco) supplemented with 10% fetal bovine serum (Integro), 1% l-glutamine (Gibco) and 1% bicarbonate (Gibco). Endpoint titrations were performed with medium containing 2% (instead of 10%) fetal bovine serum.

### Beta SARS-CoV-2 variant infection in different mouse species.

In brief, 7- to 9-week-old male SCID mice (CB-17/Icr-*Prkdc^scid/scid^*/Rj; Janvier Laboratories), male BALB/c mice (internally bred), and C57BL/6 mice (internally bred) were anesthetized with isoflurane and inoculated intranasally with 40 μL containing 10^5^ TCID_50_ SARS-CoV-2 Beta variant (day 0). Mice were housed in individually ventilated cages with a maximum of five mice per cage and monitored daily for weight changes and any clinical signs. At day 3 p.i., 9 animals were euthanized by intraperitoneal injection of 100 μL Dolethal (200 mg/mL sodium pentobarbital; Vétoquinol SA), and lungs were collected and viral RNA and infectious virus were quantified by reverse transcription-quantitative PCR (RT-qPCR) and endpoint virus titration, respectively. The left lungs were fixed in 4% formaldehyde for histopathological analysis.

### Treatment regimen.

Male SCID mice were treated by oral gavage with either the vehicle (*n* = 18) or molnupiravir (*n* = 14) at 200 mg/kg or nirmatrelvir (*n* = 14) at 300 mg/kg, twice daily starting from day 0, just before infection with the Beta variant as described in the previous section. All treatments were continued for 3 consecutive days (i.e., until day 2 p.i.). Mice were monitored for appearance, behavior and weight. At day 3 pi, mice were euthanized and lungs were collected and viral RNA and infectious virus were quantified by RT-qPCR and endpoint virus titration, respectively. The left lungs were fixed in 4% formaldehyde for histopathological analysis.

### SARS-CoV-2 RT-qPCR.

Lung tissues were collected after sacrifice and were homogenized using bead disruption (Precellys) in TRK lysis buffer (EZNA total RNA kit; Omega Bio-tek) and centrifuged (10,000 rpm, 5 min) to pellet the cell debris. RNA was extracted according to the manufacturer’s instructions. RT-qPCR was performed on a LightCycler96 platform (Roche) using the iTaq universal probes one-step RT-qPCR kit (Bio-Rad) with N2 primers and probes targeting the nucleocapsid (26). Standards of SARS-CoV-2 cDNA (IDT) were used to express viral genome copies per milligram of tissue.

### Endpoint virus titrations.

Lung tissues were homogenized using bead disruption (Precellys) in minimal essential medium and centrifuged (10,000 rpm, 5 min, 4°C) to pellet the cell debris. To quantify infectious SARS-CoV-2 particles, endpoint titrations were performed on confluent Vero E6 cells in 96-well plates. Viral titers were calculated by the Reed and Muench method (27) using the Lindenbach calculator and were expressed as the TCID_50_ per milligram of tissue.

### Histology.

For histological examination, the lungs were fixed overnight in 4% formaldehyde and embedded in paraffin. Tissue sections (5 μm) were analyzed after staining with hematoxylin and eosin and scored blindly for lung damage by an expert pathologist. The scored parameters, to which cumulative scores of 1 to 3 were attributed, were the following: congestion, intra-alveolar hemorrhagic, apoptotic bodies in bronchus wall, necrotizing bronchiolitis, perivascular edema, bronchopneumonia, perivascular inflammation, peribronchial inflammation, and vasculitis.

### Ethics.

Housing conditions and experimental procedures were approved by the ethics committee of animal experimentation of KU Leuven (license P001/2021).

### Statistics.

GraphPad Prism (GraphPad Software, Inc.) was used to perform statistical analysis. Statistical significance was determined using the nonparametric Kruskal-Wallis test. *P* values of <0.05 were considered significant.
